# MMN: From Immunological Cross-Talk to Conduction Block

**DOI:** 10.1007/s10875-014-0026-3

**Published:** 2014-04-13

**Authors:** Oliver Harschnitz, Bas A. Jongbloed, Hessel Franssen, Dirk C. G Straver, W. Ludo van der Pol, Leonard H. van den Berg

**Affiliations:** 1 Department of Neurology and Neurosurgery, UMC Utrecht Brain Center Rudolf Magnus, Utrecht, 3584 CG The Netherlands; 2 Department of Translational Neuroscience, UMC Utrecht Brain Center Rudolf Magnus, Utrecht, 3584 CG The Netherlands; 3 Department of Neurology, St. Elisabeth Hospital, Tilburg, 5000 LC The Netherlands

**Keywords:** MMN, multifocal motor neuropathy, gangliosides, anti-GM1 antibodies, immune pathogenesis, conduction block, intravenous immunoglobulin

## Abstract

Multifocal motor neuropathy (MMN) is a rare inflammatory neuropathy characterized by progressive, asymmetric distal limb weakness and conduction block (CB). Clinically MMN is a pure motor neuropathy, which as such can mimic motor neuron disease. GM1-specific IgM antibodies are present in the serum of approximately half of all MMN patients, and are thought to play a key role in the immune pathophysiology. Intravenous immunoglobulin (IVIg) treatment has been shown to be effective in MMN in five randomized placebo-controlled trials. Despite long-term treatment with intravenous immunoglobulin (IVIg), which is efficient in the majority of patients, slowly progressive axonal degeneration and subsequent muscle weakness cannot be fully prevented. In this review, we will discuss the current understanding of the immune pathogenesis underlying MMN and how this may cause CB, available treatment strategies and future therapeutic targets.

## Introduction

Multifocal motor neuropathy (MMN) is a rare immune-mediated, pure motor neuropathy with a prevalence of at least 0.6 per 100,000 individuals [1]. The male:female ratio is 2.7:1 [1] and the mean age at onset is 40 years, with a range of 20–70 years [1–3]. In contrast to other immune-mediated polyneuropathies such as the Guillain-Barre syndrome (GBS) or chronic inflammatory demyelinating polyneuropathy (CIDP), onset of MMN does not occur in childhood or old age.

MMN is characterized by slowly progressive, asymmetric muscle weakness in distal limbs. The ulnar, median, radial and tibial nerves are most often affected [1, 4]. Muscles may be atrophic, and there is a striking lack of sensory symptoms. Other clinical features include muscle cramps, fasciculations, and an increase of weakness in cold conditions [4, 5]. For a more comprehensive overview of the clinical characteristics and diagnostic criteria we would like to refer to the review by Vlam et al. [4].

Persistent conduction block (CB) is the electrophysiological hallmark of the disease, and distinguishes MMN from motor neuron disease (MND) [6–8]. In contrast to patients with MND, MMN patients have a normal life expectancy and respond well to treatment with intravenous immunoglobulin (IVIg). However MMN does not necessarily follow a benign disease course, and up to 20 % of patients report relatively severe disability predominantly of the upper limbs.

The pathophysiology of MMN remains to be elucidated. Pathological studies are relatively scarce and have yielded conflicting results [9–12]. The presence of antibodies against GM1 and the favourable response to IVIg treatment support an immune mediated pathophysiology. MMN has a distinct overlap in clinical features with acute motor axonal neuropathy (AMAN), the pure motor axonal form of GBS, implying an analogy in underlying disease mechanisms. This further argues in favour of an immune mediated disease pathophysiology.

In this review, we will focus on the immune pathophysiology of MMN and on CB, and how this knowledge may help to develop novel therapeutic strategies.

## Immune Pathophysiology of MMN

### Anti-GM1 IgM Antibodies

The presence of anti-GM1 IgM antibodies has been documented in the earliest descriptions of MMN [13], and is, together with the virtually universal response to IVIg, the most important clue that MMN is a primarily inflammatory disorder. Prevalence studies on GM1 antibodies are complicated by methodological differences and the lack of a gold standard to measure the presence and titre of these antibodies [14]. In a recent study we found anti-GM1 antibodies in serum of at least 50 % of a large cohort of patients with MMN using a very specific ELISA protocol [1]. With the exception of a minor subset of patients with anti-GM2 and anti-GD1b IgM antibodies, both shown to be cross-reactive with GM1 in absorption studies, we were unable to corroborate previously reported associations with other anti-ganglioside antibodies [4]. It is not understood why there is a lack of anti-GM1 antibodies in almost half of MMN patients. First of all, this could be due to methodological issues, where a low sensitivity could hamper detection of antibodies [14]. Furthermore it could be that some, or even all, MMN patients harbour antibodies against other, as of yet unknown, antigens [15–17]. This will be discussed in more detail further on.

Anti-GM1 IgM antibodies probably belong to the natural antibody repertoire that are secreted by a specific subset of innate B cells. In patients with MMN it may well be that anti-GM1 IgM antibodies are produced by a single or very few B-cell clones as shown by their restricted immunoglobulin light chain use (Cats et al., unpublished data) and the association of MMN with IgM monoclonal gammopathy (Vlam et al., unpublished data). High titres of these antibodies are associated with MMN and are rare in patients with lower motor neuron disease or GBS [1, 14, 18]. The titres of anti-GM1 IgM antibodies correlate with their complement-activating capacity in vitro [19, 20] and with the severity of muscle weakness [1].

Assuming a pathogenic role of anti-GM1 IgM antibodies, the selective involvement of motor axons is not fully understood. The most straightforward hypothesis is that GM1 is selectively expressed in motor nerves, implying an increased vulnerability of motor axons to anti-ganglioside antibodies. Experimental studies have addressed this issue, and one such study found GM1 to be more abundant in the ventral roots compared to dorsal roots [21], but this has not been corroborated in other studies [22, 23]. However, from these studies it is also apparent that GM1 is, to some degree, also present in unaffected nerves. For this reason, even if there would be a difference in GM1 expression in nerves, this cannot be the sole reason for motor neuron selectivity in MMN.

Other explanations include slight differences in the molecular composition of gangliosides between motor and sensory nerves [22, 24] or differences in the association with other glycolipids or the density of these structures on the axolemma of motor and sensory nerves [25–27]. Findings in AMAN support differences in GM1 expression between sensory and motor nerves. Furthermore, serum from AMAN patients containing GD1a-specific IgG antibodies show preferential binding to motor axons, despite a lack of quantitative differences in GD1a ganglioside expression [22, 28]. Differences in the fatty acid chain length of the ceramide portion of gangliosides [22] or differences in cholesterol content in motor and sensory axons could induce changes in the expression of specific GM1 epitopes and thereby determine antibody-binding opportunities. Finally, it may be that anti-GM1 IgM antibodies bind to both motor and sensory nerves, but that sensory nerves are less vulnerable to damage due to differences in biophysical properties. This is supported by the finding that slight changes in vibration sense occur in patients with MMN with longer disease duration [1].

### Pathogenic Effects Mediated by Anti-GM1 Antibodies

**Fig. 1:**
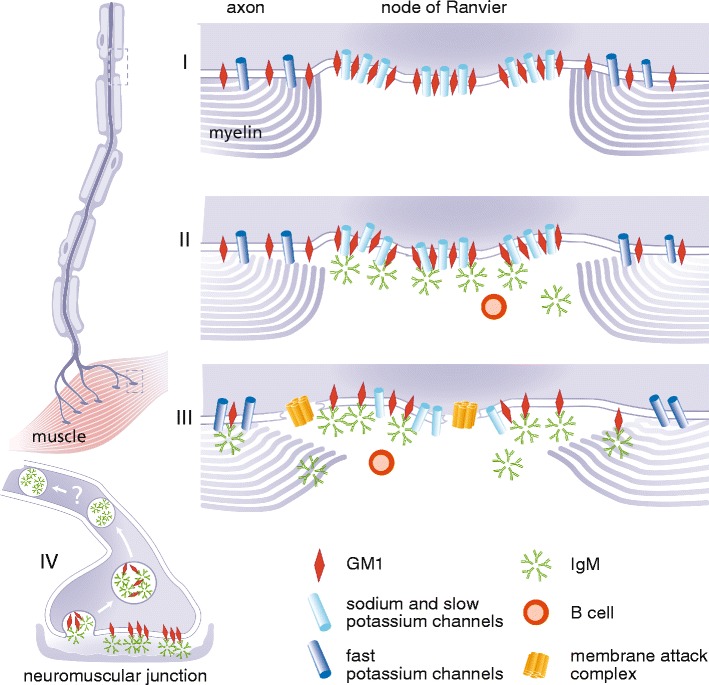
Schematic model of putative disease mechanisms in MMN. Anti-GM1 IgM antibodies may trigger direct and complement dependent damage to axons. In the normal physiological situation the node of Ranvier is characterized by clusters of ion channels, held together by GM1 and other lipids in so called lipid rafts (I). These voltage gated sodium and slow voltage gated potassium channels, together with fast voltage gated potassium channels in the paranodal region, maintain normal saltatory conduction. Paranodal myelin is attached to the axon by GM1. Activated B cells (plasma cells) produce the pentameric IgM antibodies that bind to GM1, possibly to heteromeric complexes containing GM1, cholesterol and galactocerebroside (not depicted in this figure) (II). The binding of these anti-GM1 antibodies can lead to the first signs of demyelination and possible dysfunction of the voltage gated sodium and slow voltage gated potassium channels. Once there is binding of anti-GM1 antibodies to GM1 the classical complement pathway is activated, and deposition of complement factors such as membrane attack complex (MAC) can take place (III). While focal demyelination continues, deposition of MAC may lead to further disruption of the Schwann-cell-axolemma junctions, displacement of ion-channel clustering and disturb membrane integrity at the (para)nodal region. Loss of fast voltage gated potassium channels through severe demyelination in the paranodal region can lead to leakage of potassium and subsequent hyperpolarization. At the site of the neuromuscular junction (NMJ) (IV), anti-ganglioside antibodies are rapidly internalised after binding, thus preventing the activation and deposition of complement factors. It is as of yet unknown whether retrograde transportation into the proximal part of the axon plays a role in the pathogenesis of MMN

Anti-GM1 IgM antibodies may trigger direct and complement dependent damage to axons (see Fig. 1). Experimental models of AMAN have illustrated that interaction of anti-ganglioside antibody with complement is a crucial step in the pathogenesis. Complement-activating properties of anti-GM1 antibodies were associated with the occurrence of weakness in the rabbit model of AMAN [29]. Complement activation leads to structural alterations in the paranodal region and subsequent disruption of ion channel integrity [30] through activation of the classic complement pathway that results in formation of membrane attack complex (MAC). MAC is a porin that compromises membrane integrity [30, 31], allowing uncontrolled ion flux. This may eventually lead to calpain activation and subsequent paralysis of the endocytic machinery of the cell and disruption of sodium channels, allowing further binding of antibodies to the axolemma [32]. Interestingly there is an increased vulnerability to complement mediated injury of distal nodes of Ranvier in motor axons compared to proximal nodes [31], possibly explaining the distal dominant pattern of weakness as observed in patients. The use of complement inhibitors abrogates the anti-ganglioside antibody mediated damage in animal models, providing more evidence for complement dependent pathology [31, 33, 34]. Although in vitro or animal models for MMN are not available, several studies have shown that anti-GM1 IgM antibodies in sera from patients with MMN also activate the classic complement pathway, and that their complement-activating potential correlates with antibody titres [19, 35] and weakness (Vlam et al., unpublished data). High innate activity of the classical complement pathway and efficient activation of this pathway correlates with both more severe axonal loss and weakness in MMN patients (Vlam et al., unpublished data).

### Blood Nerve Barrier Disruption

The peripheral nerves are protected by the blood-nerve barrier (BNB) from inflammatory cells and antibodies. The large molecular size of anti-GM1 IgM antibodies (900 kD) [36] suggests that BNB disruption plays an important role in MMN pathogenesis. Rare pathological studies have reported perivascular lymphocytic infiltration in the endoneurial microvessels of the BNB [9–12]. The presence of circulating cytokines, such as VEGF, TNF-α, and IL-1β, appears to be linked to dysfunction of the BNB in MMN patients [37]. A recent study using an in vitro BNB model, consisting of conditionally immortalised human BNB-derived endothelial cells, has suggested that VEGF is the main effector molecule linked to the pathogenesis of the BNB breakdown [38]. Although serum concentrations of VEGF did not differ between MMN patients and healthy controls, the addition of a neutralising anti-VEGF antibody to the MMN sera resulted in restoration of BNB function [38]. VEGF secretion by endothelial cells was increased after incubation with MMN sera, suggesting the effect of VEGF occurred via an autocrine mechanism.

### Pre-synaptic Motor Nerve Terminal Uptake

In addition to gangliosides in the paranodal region of motor neurons, several studies suggest that auto-antibodies may also target antigens in the presynaptic membrane of the neuromuscular junction (NMJ). Gangliosides at this site are likely targets since their density at the synaptic membrane is relatively high [39], and there is no BNB to offer protection. Gangliosides at the presynaptic membrane of the NMJ undergo recycling through endosomal pathways [40].

Despite their physiological synaptic abundance, the role of complex gangliosides at the NMJ is unclear. They seem dispensable for transmitter release and more specifically electrophysiological signal propagation at the neuromuscular junction. This is supported by normal electrophysiological properties in transgenic mice lacking all gangliosides except for GM3 [41, 42]. Studies using in vitro incubation of mouse hemidiaphragm preparations with anti-ganglioside antibodies showed nerve terminal damage and electrophysiological investigations revealed block of signal transmission [43]. It must be noted that this process was complement-dependent. The relevance of this mouse-model for MMN pathogenesis is unknown. A recent study by Fewou et al. [40] showed that rapid internalisation of anti-ganglioside antibodies at the presynaptic membrane prevented complement-mediated cytotoxicity. This protective mechanism is not available at the nodes of Ranvier and paranodal regions, and could thus explain why dysfunction of these structures underlies MMN pathogenesis. In contrast to the local (para)nodal neurotoxic effect, the effect of antibody internalisation and retrograde transport is not fully understood. Further studies are needed to determine the possible toxic effect of antibody uptake and retrograde transport [40].

### Origin of Pathogenic Antibodies

The mechanisms of B cell activation leading to elevated anti-GM1 IgM titres in MMN are yet to be established. Unlike AMAN, associations with preceding microbial or viral infections leading to the production of cross-reactive anti-ganglioside antibodies through molecular mimicry have not been reported in MMN [44]; the slowly progressive, chronic nature of MMN makes studying preceding infections more challenging compared to the more acute disease course of for example AMAN. Alternatively, monoclonal B-cell proliferation is suggested by an increased frequency of IgM monoclonal gammopathy, which is seen more frequently in patients compared to healthy controls (Vlam et al., unpublished data). However there are no studies on the cellular content of bone marrow of patients with MMN.

MMN lacks features of classic autoimmune disease, with a lack of response to corticosteroid treatment and a male predominance. Nevertheless, we found a slightly increased frequency of autoimmune disease in MMN patients as compared to controls, suggesting shared pathogenic mechanisms [45]. The HLA-DRB1*15 haplotype was increased among Dutch patients with MMN, similar to patients with multiple sclerosis and female patients with chronic inflammatory demyelinating polyneuropathy (CIDP) [30]. Since there is no evidence that T-cells play a role in MMN pathogenesis, the association with HLA-DRB1*15 may reflect an increased propensity for the production of autoantibodies, as has been suggested for a number of other disorders [46].

### Anti-GM1 IgM Negative Cases: Antibodies Against Other Antigens?

Approximately half of all patients with MMN lack elevated titres of anti-GM1 IgM antibodies in enzyme-linked immunosorbent assay (ELISA) [1, 14]. It is unknown whether these patients have low titres of anti-GM1 IgM antibodies that are undetectable with ELISA, or whether they have antibodies against other, as of yet unidentified, antigens. The clinical characteristics of patients with and without anti-GM1 antibodies do not differ, and treatment response is seen in seropositive as well as in seronegative patients [20, 47], although weakness and disability are somewhat more pronounced in seropositive cases on a group level [1].

Antibodies against NS6S (a disulphated heparin disaccharide) have been found in patients with chronic inflammatory neuropathies, and possibly in MMN [48]. However, the relevance of NS6S as an antigen in MMN pathogenesis remains to be corroborated. Earlier studies have suggested that heteromeric complexes including GM1 facilitate increased binding of GM1-specific antibodies. Heteromeric complexes are structurally distinct glycolipids that interact to form new molecular shapes capable of enhancing recognition by antibodies [49]. Although we did not find antibodies to combinations of gangliosides in sera from patients with MMN [1, 49], anti-GM1 IgM antibodies have been shown to bind more strongly to a lipid mix of GM1, galactocerebroside and cholesterol (GGC) [15]. These results have recently been reproduced using both combinatorial glycoarray and ELISA, suggesting that GM1/galactocerebroside complexes are specific antigens in MMN [16, 17].

The idea that heteromeric complexes, where accessory lipids besides GM1, play a crucial role in the binding of GM1-specific IgM antibodies and that possible interplay between glycolipids in the bilipid membrane of axons can substantially increase antibody binding is of great interest in MMN. On a structural level there are three mechanisms in which heteromeric complexes are thought to alter anti-ganglioside antibody binding; through conformational modulation, steric hindrance and the generation of neo-epitopes [49]. The formation of neo-epitopes by structural alteration is yet to be proven at a molecular level. However, it has been shown that cholesterol can induce changes in ligand binding to glycolipids, by inducing a tilt in the glycolipid receptor headgroup [50]. It is therefore not unthinkable that galactocerebroside and cholesterol interact with GM1 in such a way that its receptor affinity is significantly enhanced. On the one hand these recent studies provide hope that the ELISA methodology and subsequent sensitivity can be further increased, while on the other hand it offers new insights into anti-ganglioside antibody induced pathogenesis.

### Relationship Pathophysiology and Symptoms

How GM1-specific IgM antibody mediated immune pathophysiology eventually leads to conduction block and muscle weakness is not fully understood. Proposed mechanisms of conduction block are threefold, namely through paranodal or segmental demyelination, abnormal resting membrane potential, and finally disruption of the clustering of nodal sodium channels and GM1 in lipid rafts [51, 52]. Experimental models suggest that binding of anti-GM1 IgG [53] to GM1 in the axolemma causes blocking and disruption of sodium channels. Sodium channel clustering is crucial for nerve conduction since it safeguards the safety factor for generating action potentials and thus propagation of the signal.

Electrophysiological studies have shown signs of dysfunction at the nodes of Ranvier, with resting membrane changes around sites of CB. Through paranodal disruption edema and GM1-antibody complexes may preclude optimal functioning of the electrogenic Na^+^/K^+^ ATPase to correct for continuous Na^+^ influx resulting in permanent focal depolarization. Distal of the CB, permanent hyperpolarization is seen probably due to overactivation of Na^+^/K^+^ ATPase in order to remove the Na-accumulation; since, per cycle, the pump removes 3 Na^+^ from the axon in exchange for 2 K^+^, increased activity results in a more negative membrane potential. One hypothesis is that at sensory nerves the density of Na^+^/K^+^ ATPase is higher and their cumulative function can correct for the ion fluxes so no conduction block is seen [51, 54–57]. The relatively rapid response to IVIg treatment in all probability does not reflect remyelination, and is more likely due to a decrease in persistent Na^+^ current [58].

GM1 also plays a role in axo-glial integration and binding of anti-GM1 IgM disrupts this resulting in demyelination and finally to axonal degeneration.

Paresis of muscles innervated by affected nerves is the end result. However, on nerve conduction studies more conduction abnormalities are seen than can be expected from a clinical point of view [59]. It is not clear which mechanisms can explain this discrepancy. One possibility is that findings from nerve conduction studies precede clinic symptoms, but long-term follow up studies to evaluate this are yet to be performed.

## Treatment

The only effective treatment options for MMN are intravenous immunoglobulin (IVIg) and possibly subcutaneous immunoglobulin (SCIg) [3, 4, 60, 61]. The response rate to IVIg is around 70–94 % [60, 62, 63]. Plasma exchange and corticoids, effective in other immune mediated neuropathies like CIDP, are not effective and can even lead to a clinical deterioration. The use of high-dose cyclophosphamide, a potent B cell suppressor, could reduce symptoms, however, the risk of adverse events including neoplasms precludes long-term usage [3, 4]. Add-on therapy with immunosuppressive drugs has been reported, but has not been assessed in randomized trials with the exception of mycophenolate mofetil, which did not alter clinical functioning or weekly IVIg dosage [64].

IVIg exerts a range of immune modulatory mechanisms, but it has not been established which are most relevant in MMN. Since complement most probably plays an important role in MMN pathogenesis, complement attenuation by IVIg could be of great significance. IVIg may prevent C3 deposition in nerves and also reduce serum concentration of key components of the classical complement pathway such as C1q and C4 [19, 35], which are crucial in the pathogenesis of immune mediated neuropathies [30].

Additional complement-modulating treatment strategies may therefore be worth pursuing. Eculizumab, a monoclonal antibody against C5 that prevents formation of membrane attack complex (MAC), was safe in a small-scale pilot study, but more detailed studies are needed to assess efficacy [65]. Nafamostat mesilate, a serine protease inhibitor with complement attenuating properties, was tested in animal models for AMAN. It prevented complement deposition and (subsequently) sodium channel disruption [66]. Up to this point it has not been tested in patients with MMN.

Another interesting approach is depletion of auto-reactive B-cells. Beneficial effects of Rituximab, a monoclonal antibody against a B-cell specific antigen CD20, have been reported in some studies, but most have not shown a sustainable effect [67, 68]. Efficacy of this biological may be restricted to patients with specific genotypes of the IgG Fc receptor expressed on NK-cells, i.e. FcγRIIIA (FcγRIIIA-V/V1158), as was suggested by a recent study in patients with a polyneuropathy associated with IgM monoclonal gammopathy [69].

### Other Future Treatment Strategies

Tailor-made IVIg therapy for patients with MMN may be another approach to improve treatment efficacy. Initial treatment with IVIg typically consists of a dose of 2 g per kg bodyweight in a 2 to 5 day course. Maintenance treatment of 0.4–1.0 g per kg bodyweight every 1 to 4 weeks is required in most patients to maintain improved muscle strength. Peak concentration of IgG is detected immediately after infusion, and rises to a 4-fold of normal levels [70]. There are nevertheless large differences in IgG pharmacokinetics between patients. In patients with GBS, lower peak concentrations after IVIg administration were an independent predictor of unfavourable outcome [71]. A recent study of IgG pharmacokinetics in 23 MMN patients during first IVIg administration showed a similar trend. Higher IgG elevation on day 1 was associated with a response to IVIG [20]. SCIg has been proposed as an alternative to IVIg. A drawback is that currently SCIg treatment is only possible using small volumes and that patients will have to use multiple infusions at multiple sites. The use of recombinant hyaluronidase (rHuPH20) allows a 10–15-fold volume increase of subcutaneously delivered IgG and this may be a useful addition for future therapy [72].

Besides immune modulatory therapies, improving conduction properties of nerves may be another approach. 3,4 Diaminopyridine is a broad-spectrum inhibitor of fast voltage-activated K^+^ (Kv) channels and improved action potential propagation in in vitro models of demyelination. In previous studies, it had no significant change on clinical outcome or on conduction blocks in small patient groups with CIDP, MMN and GBS and this was confirmed in a double blind placebo controlled study [73, 74]. The duration of administration lasted only several days and longer schedules may be worth considering in future trials.

## Concluding Remarks

MMN is a rare pure motor neuropathy characterized by predominantly distal, asymmetric limb weakness with CB as an electrophysiological hallmark. Despite the fact that the pathogenesis of MMN is yet to be fully understood, there are clear signs suggesting an immune-mediated pathogenesis. A beneficial response to IVIg underlines this, as does the presence of anti-GM1 IgM antibodies in more than half of MMN patients. The origin of these antibodies together with the mechanism how anti-GM1 IgM antibodies exert their neurotoxic effect at a molecular level remains unclear. However functional studies have shown complement activating potential of patient sera harbouring anti-GM1 IgM antibodies, and models of AMAN have highlighted the importance of complement dependent pathology. Future research will be required to determine the exact binding epitope of anti-GM1 antibodies, and if heteromeric complexes containing GM1 and other lipids in any way influence the binding affinity of these or other unknown antibodies. Regardless of the underlying molecular mechanism, it is the phenomenon of persistent conduction block and ultimately axonal degeneration that results in muscle weakness. To date the only available treatment is IVIg, which is efficacious in most patients. However long term disability remains a problem, as muscle weakness is slowly progressive in the majority of patients despite treatment. A tailor-made approach to modify IVIg therapy seems to be the best way to improve current outcome for MMN patients. Other modes of therapy could be improving the conduction properties of nerves or attenuating complement activation. Further studies are necessary to unravel the exact underlying disease mechanism and uncover novel therapeutic targets for this chronic and potentially debilitating disorder.
